# Complete Genome Sequence of Potential Probiotic *Lactobacillus* sp. HFC8, Isolated from Human Gut Using PacBio SMRT Sequencing

**DOI:** 10.1128/genomeA.01337-15

**Published:** 2015-11-19

**Authors:** Madhu Kumari, Mohit Kumar Swarnkar, Sanjay Kumar, Anil Kumar Singh, Mahesh Gupta

**Affiliations:** a Department of Food Neutraceuticals and Quality Control, CSIR, Institute of Himalayan Bioresource Technology, Palampur, India; b Department of Biotechnology, CSIR, Institute of Himalayan Bioresource Technology, Palampur, India

## Abstract

We report a 3.07-Mb complete genome sequence of a lactic acid bacterium, *Lactobacillus* sp. HFC8. The gene-coding clusters are predicated for probiotic characteristics, like bacteriocin production, cell adhesion, bile salt hydrolysis, lactose metabolism, autoaggregation, and tolerance to oxidative stress.

## GENOME ANNOUNCEMENT

The genus *Lactobacillus* is a group of rod-shaped, Gram-positive, non-spore-forming bacteria that belongs to the family *Lactobacillales* of phylum *Firmicutes* (1). Members of this genus are usually found in fermented foods and gastrointestinal tracts of humans and animals (2, 3). *Lactobacillus* sp. strain HFC8 was isolated from fecal samples of healthy native individuals living in the Village Kandi district Kangra, Himachal Pradesh, India (32°08′50.36″N, 76°31′37.37″E, 1,508 meters above sea level). On the basis of 16S rRNA sequencing, it was found to be closely related to *Lactobacillus plantarum* subsp. *plantarum* ATCC 14917^T^, *Lactobacillus pentosus* JCM 1558^T^, and *Lactobacillus paraplantarum* DSM10667^T^. Strain HFC8 belongs to a taxonomic group that is indistinguishable by 16S rRNA sequencing only (4). Strain HFC8 is a good probiotic candidate as it shows high tolerance to simulated gastrointestinal fluids and antibacterial activity against *Micrococcus leuteus*, *Escherichia coli*, *Klebsiella pneumoniae*, *Staphylococcus aureus*, *Salmonella typhi*, and *Pseudomonas aeruginosa*, major criteria for probiotic selection (5, 6).

Owing to the potential of HFC8 as a probiotic, the complete genome was sequenced. The genomic DNA was isolated using the phenol chloroform-isoamyl alcohol extraction procedure from 24-h-old cultures of *Lactobacillus* sp. HFC8 in MRS broth flasks incubated at 37°C. The quantity and quality of genomic DNA were determined by a NanoDrop 2000 UV-Vis spectrophotometer (Thermo Scientific, USA) and Qubit 2.0 fluorometer (Invitrogen, USA). The quality of genomic DNA sheared using Covaris g-tubes was checked with a Bioanalyzer DNA 12000 chip (Agilent Technologies, USA). The genomic DNA library with a 10-kb DNA insert size was prepared using a PacBio SMRTbell library preparation kit v1.0 as described (7). The library was quantified using a Qubit 2.0 fluorometer and sequenced on a PacBio RS II system on two SMRT cells employing a P5 polymerase and C3 chemistry combination (P5−C3) with a 180-min movie. The SMRT cells produced 1,315,264,242 bases generated through 125,649 reads (*N*_50_ size 17,277 bp and mean read length 10,467 bp). All generated subreads were assembled *de novo* using an RS hierarchical genome assembly process (HGAP) protocol version 3.0 in SMRT analysis version 2.2.0 (Pacific Biosciences) that yielded a gapless completed circular chromosome sequence, with 210-fold coverage. Functional annotation was performed on the Rapid Annotation using Subsystems Technology (RAST) server (8).

The one complete circular chromosome was 3,067,675 bp in size with an estimated GC content of 44.33% and 10 plasmids, totaling 3,405,709 bp. The RAST predicted 3,447 protein-coding genes (CDSs) and 85 RNA genes, assigned through 334 RAST subsystem categories. Based on RAST annotation, strain HFC8 is closely related to *Lactobacillus plantarum* WCFS1 (score 540) and *Lactobacillus plantarum* subsp. *plantarum* ATCC 14917 (score 515).

Analysis of the genome revealed that strain HFC8 contains genes for bacteriocin production, cell adhesion, bile salt hydrolysis, autoaggregation, lactose metabolism, and tolerance to oxidative stress. These features may enable it to colonize in human gut and exert beneficial effects. The genome sequence also showed the presence of genes responsible for production of exopolysaccharide, vitamins, amino acids and l-asparaginase, suggesting its applications in the food and pharmaceutical industries (9, 10).

### Nucleotide sequence accession numbers.

The complete genome sequence of *Lactobacillus plantarum* HFC8 has been deposited at DDBJ/EMBL/GenBank under the accession numbers CP012650 to CP012660.
